# Potentially inappropriate prescribing in two populations with differing socio-economic profiles: a cross-sectional database study using the PROMPT criteria

**DOI:** 10.1007/s00228-015-2003-z

**Published:** 2016-01-28

**Authors:** Janine A. Cooper, Frank Moriarty, Cristín Ryan, Susan M. Smith, Kathleen Bennett, Tom Fahey, Emma Wallace, Caitriona Cahir, David Williams, Mary Teeling, Carmel M. Hughes

**Affiliations:** Clinical and Practice Research Group, School of Pharmacy, Queen’s University Belfast, 97 Lisburn Road, Belfast, BT9 7BL Northern Ireland; HRB Centre for Primary Care Research, Division of Population Health Science, Royal College of Surgeons in Ireland, 123 St Stephen’s Green, Dublin 2, Ireland; School of Pharmacy, Royal College of Surgeons in Ireland, 123 St Stephen’s Green, Dublin 2, Ireland; Department of Pharmacology & Therapeutics, Trinity Centre for Health Sciences, St. James’s Hospital, Dublin 8, Ireland; Economic and Social Research Institute, Whitaker Square, Sir John Roberson’s Quay, Dublin 2, Ireland; Department of Geriatric and Stroke Medicine, Royal College of Surgeons in Ireland, 123 St Stephen’s Green, Dublin 2, Ireland

**Keywords:** Potentially inappropriate prescribing, PROMPT, Middle-age, Polypharmacy, Explicit criteria

## Abstract

**Purpose:**

The purpose of this study is to establish the prevalence of potentially inappropriate prescribing (PIP) in middle-aged adults (45–64 years) in two populations with differing socio-economic profiles, and to investigate factors associated with PIP, using the PROMPT (PRescribing Optimally in Middle-aged People’s Treatments) criteria.

**Methods:**

A retrospective cross-sectional study was conducted using 2012 data from the Enhanced Prescribing Database (EPD), covering the full population in Northern Ireland and the Health Services Executive Primary Care Reimbursement Service (HSE-PCRS) database, covering the most socio-economically deprived third of the population in this age group in the Republic of Ireland. The prevalence for each PROMPT criterion and overall prevalence of PIP were calculated. Logistic regression was used to investigate the association between PIP and gender, age group and polypharmacy.

**Results:**

This study included 441,925 patients from the EPD and 309,748 patients from the HSE-PCRS database. Polypharmacy was common in both datasets (46.7 % in the HSE-PCRS and 20.3 % in the EPD). The prevalence of PIP was 42.9 % (95%CI 42.7, 43.1) in the HSE-PCRS and 21.1 % (95%CI 21.0, 21.2) in the EPD. Age group, female gender and polypharmacy were significantly associated with PIP in both populations (*p* < 0.05) and polypharmacy had the strongest association.

**Conclusions:**

PIP is common amongst middle-aged people with the risk of PIP increasing with polypharmacy. Differences in the prevalence of polypharmacy and PIP between the two populations may relate to heterogeneity in healthcare services and different socio-economic profiles, with higher rates of multimorbidity and associated polypharmacy in more deprived groups.

**Electronic supplementary material:**

The online version of this article (doi:10.1007/s00228-015-2003-z) contains supplementary material, which is available to authorized users.

## Introduction

As prescribing guidelines generally focus on single diseases [1], the increasing prevalence of patients with two or more chronic conditions, or multimorbidity [2], creates challenges in determining the appropriateness of prescribing. Traditionally, the focus of potentially inappropriate prescribing (PIP) has been on older people (routinely defined as aged 65 years and over) due to the complexity of prescribing for patients in this age group, including changes in pharmacokinetics and pharmacodynamics [3], and a high prevalence of multimorbidity (82 % aged ≥85 years; 65 % aged 65–84 years) [2]. Multimorbidity presents risks to patients including poor quality of life and increased healthcare services utilisation [4], and is associated with the use of multiple medications, or polypharmacy, which may increase the risk of adverse drug events or hospitalisation [5]. However, polypharmacy may be appropriate in the treatment of many conditions [6], and the challenge for prescribers is to determine the appropriateness of prescribing in the context of multiple conditions and medications while prioritising therapeutic management when there is a conflict between medications and/or illnesses. PIP may be evaluated using explicit (criterion-based) or implicit (judgement-based) tools which have been developed to optimise prescribing [7–13]. Despite approximately one third of middle-aged people (routinely defined as aged 45–64 years) living with multiple conditions, there has been a paucity of research in PIP in middle-aged adults [2]. PROMPT (PRescribing Optimally in Middle-aged People’s Treatments) represents a set of 22 explicit prescribing criteria, organised according to physiological system, which have been developed specifically for middle-aged adults [8]. This set of criteria may be applied to administrative datasets, or drug lists alone (i.e. in the absence of clinical information), to determine the prevalence of PIP in middle-aged people [8]. The criteria are similar to the Screening Tool for Older Persons’ Prescription (STOPP) [10] in mainly specifying circumstances in which a medicine may be inappropriate (co-morbidities, dosage, duration of use) rather than stating drugs to avoid in all cases as is more common in the Beers criteria [9].

The aim of this study is to establish the prevalence of PIP in middle-aged adults defined using the PROMPT criteria in two populations (Northern Ireland and the Republic of Ireland) with differing socio-economic profiles, and investigate any association between PIP and polypharmacy, gender and age group.

## Methods

### Study design and setting

This is a retrospective cross-sectional study using data from the Enhanced Prescribing Database (EPD) in Northern Ireland and the Health Services Executive Primary Care Reimbursement Service (HSE-PCRS) database in the Republic of Ireland.

### The Enhanced Prescribing Database 

The Enhanced Prescribing Database (EPD), first established in 2008, is a population-based dataset which collates information on computer-generated prescriptions issued in general practice and subsequently dispensed in a community pharmacy in Northern Ireland. Prescriptions generated in general practices contain two-dimensional (2-D) barcodes which hold all relevant information on the prescription for example, patient details [name, address, age, date of birth and Health and Social Care (HSC) number], prescriber details (general practice and prescriber) and medication information [medication(s) prescribed including dose, strength and quantity and date of issue]. Prescriptions dispensed by community pharmacies are forwarded to the data custodians, the Business Services Organisation (BSO), each month for reimbursement where the barcodes are scanned, with data from approximately 85–90 % of all prescriptions added to the EPD database [14]. Since 2010, all medicines available in Northern Ireland via the National Health Service (NHS) have been free at the point of dispensing. According to Census data for Northern Ireland, there were approximately 442,000 individuals in Northern Ireland aged between 45 and 64 years (males, 49 %; females, 51 %) in 2011 [15].

### The Health Services Executive Primary Care Reimbursement Service

The Health Services Executive Primary Care Reimbursement Service (HSE-PCRS) database records pharmacy claims for dispensed medicines that were prescribed to patients by their general practitioner (GP). Drug information on strength, quantity dispensed, dosage form and defined daily doses (DDD) is also included. Some patient demographic data are recorded such as age, gender and region. The General Medical Services (GMS) scheme is a form of public health cover that provides free health services including prescription medicines to eligible persons in Ireland (although a small monthly co-payment of €0.50 per medication was introduced in October 2010). Eligibility for the scheme is determined by means testing and a common family/household income threshold applies to people aged <70 years, with a higher income threshold for older individuals. Approximately 40.4 % of the total population of the Republic of Ireland were eligible for the scheme in 2012, while 348,025 of 1,054,300 individuals aged 45–64 years in the national population (33 %) were eligible [16, 17]. Due to the eligibility criteria, the GMS scheme over-represents socio-economically deprived individuals, particularly those aged <70 years.

## Study population

For each dataset, the study population contained all individuals aged 45 years or older on 1st January 2012. Prescribing data were included until 31st December 2012, or until the individual’s 65th birthday (month), whichever came first. In Northern Ireland, the study population included all patients who remained registered with a general practice in Northern Ireland during the study period. Patients who migrated into or from Northern Ireland, or died during the study period, were excluded from the cohort. Furthermore, only patients registered with general practices with a scan rate greater or equal to 80 % [i.e. (ratio of readable barcodes produced by the general practice in 1 month/total number of dispensed prescriptions from the general practice in 1 month)*100] were included in the study population. In the Republic of Ireland, the study population included all individuals eligible for the HSE-PCRS GMS scheme, who were dispensed a prescription during the study period. The research team had no access to patient identifiable information in either dataset. Ethical approval for the use of EPD data was obtained from the NHS Research Ethics Committees (REC), under proportionate review (REC reference 14/SW/0038). Permission was given by the data controller of the HSE-PCRS database for use if data were anonymised and analysed so that no subgroup contained fewer than five individuals, and therefore, it was not necessary to seek specific ethical approval for this analysis.

## Data extraction

Data were extracted for the study period between 1st January 2012 and 31st December 2012. In the EPD dataset, medications were extracted using British National Formulary (BNF) codes, whereas in the HSE-PCRS database, World Health Organisation (WHO) Anatomical Therapeutic Chemical (ATC) codes were used. Dispensing data prior to the study start date were required to determine previous medication history for some criteria [for example, Tricyclic antidepressants (TCAs) (e.g. amitriptyline, nortriptyline) should not be used as first-line in treatment of depression]. Therefore, a lead-in period of 3 months was required for all patients (data were extracted between 1st October 2011 and 31st December 2012). A description of the analytical approach used to apply various criteria is included in Supplementary file 1.

## Outcomes

Patients were categorised as having received, or not having received, any of the PIPs listed in the 22 criteria [8]. The primary outcome was the overall prevalence of PIP, defined as having any one of the 22 PROMPT criteria. Secondary outcomes were the prevalence of individual PROMPT criteria, and the association between presence of any PIP (binary variable), polypharmacy and gender (male/female). These factors were assessed as they have been shown to be associated with PIP in older people in previous research [18]. The association between increasing age groups (45–49, 50–54, 55–59 and 60–64 years) was also investigated as a secondary outcome. Polypharmacy was assessed by calculating the number of different medication classes which were dispensed at least three times to each individual during the study period. Medication classes were defined by BNF sections in the EPD and five-character ATC codes in the HSE-PCRS. Although there is no universally accepted definition of the term polypharmacy, the conventional definition of four or more medications was used in this study [19].

## Statistical analysis

A percentage estimate with 95 % confidence intervals (CI) for the overall prevalence of PIP for all the PROMPT criteria and the prevalence of PIP defined by each individual PROMPT criterion were calculated. These estimates represent the number of individuals exposed to a PIP as a proportion of all individuals in each dataset (i.e. all those from the included populations who were dispensed a prescription during 2012). Adjusted logistic regression analyses were used to calculate odds ratios (OR) and 95 % CI to investigate any association between any PIP and polypharmacy, gender and age groups. Data extraction from the EPD was performed in MySQL and data were incorporated into STATA version 11.0 [StataCorp 2005, College Station, TX, USA] for analyses. Analyses on the HSE-PCRS database were performed using SAS 9.2 (SAS Institute Inc., Cary, NC, USA).

## Results

### Descriptive statistics

This study included 441,925 patients from the EPD and 309,748 patients from the HSE-PCRS database. Descriptive statistics for these two populations are shown in Table 1. Polypharmacy levels were high, with 46.7 and 20.3 % of individuals receiving ≥4 different drugs classes in the HSE-PCRS database and EPD database, respectively.

**Table 1:** Participant demographics

	EPD^a^ (N = 441,925)		HSE-PCRS^b^ (*N* = 309,748)	
	*n*	%	*n*	%
Female	218,498	49.4	165,588	53.5
Polypharmacy	89,631	20.3	144,485	46.7
Age group				
45–49 years	125,979	28.5	67,424	21.8
50–54 years	114,585	25.9	74,298	24.0
55–59 years	97,740	22.1	73,440	23.7
60–64 years	103,621	23.5	94,586	30.5
	Mean	SD	Mean	SD
Age (years)	54.1	6.01	55.3	5.84

^a^Enhanced Prescribing Database, covering the full middle-aged population of Northern Ireland

^b^Health Service Executive Primary Care Reimbursement database, covering the most socioeconomically deprived third of the middle-aged population of the Republic of Ireland

### Prevalence of overall PIP and criterion-specific PIP

The overall prevalence of PIP was 42.9 % (95 % CI 42.7, 43.1) in the Republic of Ireland and 21.1 % (95 % CI 21.0, 21.2) in Northern Ireland (Table 2). Prevalence estimates were calculated for each individual PROMPT criterion (and are included in Supplementary file 2) and a number of criteria were common in both populations (Table 3). Strong opioids without co-prescription of an osmotic or stimulant laxative was the most prevalent instance of PIP in the EPD (prevalence 6.9 %), followed by prescription of proton pump inhibitors (PPIs) above maintenance dosage for greater than 8 weeks (6.9 %). In the HSE-PCRS database, these criteria were the second and first most common criteria, found in 13.9 and 17.7 % of individuals respectively. The next most prevalent PIP was the same in both populations, with a benzodiazepine being prescribed long-term (for >4 weeks) to 2.9 % of individuals in the EPD and 8.5 % of those in the HSE-PCRS database. The long-term use of non-benzodiazepine hypnotics (Z-drugs) was fourth (8.3 %) and fifth (2.5 %) most prevalent in the HSE-PCRS and EPD populations, respectively. The other most common PIP differed between the two populations, with first generation anti-histamines being prescribed for greater than 7 days for 2.5 % of EPD patients and only 0.5 % of HSE-PCRS patients, and non-steroidal anti-inflammatory drugs (NSAIDs) being prescribed for greater than 3 months to 5.0 % of individuals in the HSE-PCRS database and 1.4 % of those in the EPD.

**Table 2:** Summary PIP prevalence estimates

	EPD^a^		HSE-PCRS^b^	
	*n*	% (95 % CI)	*n*	% (95 % CI)
Overall PIP prevalence	93,319	21.1 (21.0, 21.2)	132,813	42.9 (42.7, 43.1)
Number of PIPs				
1 PIP	55,960	12.7 (12.6, 12.8)	71,462	23.1 (22.9, 23.2)
2 PIPs	22,125	5.0 (4.9, 5.1)	33,403	10.8 (10.7, 10.9)
≥3 PIPs	15,234	3.4 (3.4, 3.5)	27,948	9.0 (8.9, 9.1)
Duplicate drug class prevalence	25,209	5.7 (5.6, 5.8)	35,104	11.3 (11.2, 11.4)

^a^Enhanced Prescribing Database, covering the full middle-aged population of Northern Ireland

^b^Health Service Executive Primary Care Reimbursement database, covering the most socioeconomically deprived third of the middle-aged population of the Republic of Ireland

**Table 3:** Most common instances of PIP and duplication of drug classes

	EPD^a^		HSE-PCRS^b^	
PROMPT criterion	*n Rank*	% (95 % CI)	*n Rank*	% (95 % CI)
Strong opioids should not be prescribed without the co-prescribing of at least one osmotic or stimulant laxative	30,679 *1st*	6.9 (6.9, 7.0)	43,041 *2nd*	13.9 (13.8, 14.0)
Proton pump inhibitors (PPIs) should not be prescribed at doses above the recommended maintenance dosage for >8 weeks.	30,367 *2nd*	6.9 (6.8, 6.9)	54,762 *1st*	17.7 (17.5, 17.8)
Benzodiazepines should not be used for >4 weeks	12,630 *3rd*	2.9 (2.8, 2.9)	26,395 *3rd*	8.5 (8.4, 8.6)
First-generation antihistamines should not be used as first-line agents for >7 days.	11,098 *4th*	2.5 (2.5, 2.6)	1556 *11th*	0.5 (0.5, 0.5)
Non-benzodiazepine hypnotics should not be used for >4 weeks	10,875 *5th*	2.5 (2.4, 2.5)	25,611 *4th*	8.3 (8.2, 8.4)
Non-steroidal anti-inflammatory drugs (NSAIDs) should not be used for >3 months	6284 *7th*	1.4 (1.4, 1.5)	15,488 *5th*	5.0 (4.9, 5.1)
Duplicate drug classes				
Opioids	16,356 *1st*	3.7 (3.6, 3.8)	12,523 1st	4.0 (4.0, 4.1)
Benzodiazepines	5089 *2nd*	1.2 (1.1, 1.2)	10,539 3rd	3.4 (3.3, 3.5)
NSAIDs	1656 3rd	0.4 (0.4, 0.4)	11,653 2nd	3.8 (3.7, 3.8)

^a^Enhanced Prescribing Database, covering the full middle-aged population of Northern Ireland

^b^Health Service Executive Primary Care Reimbursement database, covering the most socioeconomically deprived third of the middle-aged population of the Republic of Ireland

Analysis of duplication of drug classes within the same month in the EPD found the prevalence was 5.7 % (95 % CI 5.6, 5.8) and opioids, benzodiazepines and NSAIDs were the most commonly implicated drugs (Table 2). The prevalence of duplication in the HSE-PCRS database was 11.3 % (95 % CI 11.2, 11.4) and opioids were most likely to be duplicated, followed by NSAIDs and benzodiazepines. Duplication of stimulant laxatives occurred in 0.1 % of the EPD population (642 individuals); however, there were no instances of this in the HSE-PCRS database.

### Association of PIP with polypharmacy, gender and age

Adjusted logistic regression analysis was performed in each population to determine factors associated with having PIP (Table 4). Age group, gender and polypharmacy were all significantly associated with having PIP in the univariate logistic regression in both populations (*p* < 0.05) and odds ratios were of similar orders of magnitude. After controlling for age group and gender, the adjusted odds ratio (OR) for having PIP in those with polypharmacy compared to no polypharmacy was 11.99 (95 % CI 11.78, 12.20) in the EPD population and 7.96 (95 % CI 7.83, 8.09) in the HSE-PCRS population. Female gender was also associated with increased odds of having a PIP (adjusted OR 1.51 and 1.12 in the EPD and HSE-PCRS database, respectively). In the univariate analysis, odds of PIP increased with each 5 year increase in age group; however, once polypharmacy and gender were adjusted for, odds were higher and approximately equal across the three older age groups compared to the reference group of 45–49 years.

**Table 4:** Logistic regression analyses for having any PIP criteria by dataset

	Unadjusted OR (95 % CI)		Adjusted OR (95 % CI)	
	EPD^a^	HSE-PCRS^b^	EPD^a^	HSE-PCRS^b^
Polypharmacy (vs none)	12.11 (11.90, 12.31)	8.05 (7.92, 8.18)	11.99 (11.78, 12.20)	7.96 (7.83, 8.09)
Female (vs male)	1.51 (1.48, 1.53)	1.15 (1.13, 1.17)	1.51 (1.48, 1.53)	1.12 (1.10, 1.14)
Age group (years)				
45–49 (reference)	1	1	1	1
50–54	1.31 (1.28, 1.34)	1.31 (1.28, 1.34)	1.08 (1.05, 1.10)	1.08 (1.04, 1.09)
55–59	1.59 (1.55, 1.62)	1.61 (1.58, 1.64)	1.08 (1.06, 1.11)	1.09 (1.07, 1.18)
60–64	1.85 (1.82, 1.89)	1.79 (1.75, 1.83)	1.07 (1.04, 1.09)	1.07 (1.04, 1.09)

^a^Enhanced Prescribing Database, covering the full middle-aged population of Northern Ireland

^b^Health Service Executive Primary Care Reimbursement database, covering the most socioeconomically deprived third of the middle-aged population of the Republic of Ireland

## Discussion

### Overall prevalence of PIP

Using the PROMPT criteria, this study has shown that PIP is common among middle-aged people (42.9 % in the HSE-PCRS database in the Republic of Ireland and 21.1 % in the EPD in Northern Ireland). The difference in the overall prevalence of PIP between these two neighbouring populations may be explained by a number of factors including differences in the socio-economic profile of the included populations and heterogeneity in healthcare services between the North and Republic of Ireland. The included populations are not directly comparable, but this work allowed us to explore the prevalence of PIP across two populations with different healthcare systems. In Northern Ireland, computer-generated prescriptions issued by GPs are free of charge, and therefore, the dataset is representative of the middle-aged national population. In the Republic of Ireland, the HSE-PCRS cohort contained middle-aged people with an income level below the eligibility threshold for the GMS scheme. Therefore, the HSE-PCRS cohort represents a more socio-economically deprived population than in the EPD dataset.

If the higher prevalence of PIP observed in the HSE-PCRS database is related to socio-economic deprivation, this relationship could be driven by a number of factors. Lower socio-economic status and health literacy can have an adverse effect on the quality of patient-doctor communication and the degree of patient involvement in shared decision making, which may potentially impact on the quality of care and the risk of PIP [20, 21]. Poorer health has been reported in socio-economically deprived areas [22], with an increased prevalence of long-term conditions including depression, anxiety, pain and coronary heart disease [23]. A large cohort study using primary care data for nearly 300,000 patients aged ≥30 years from the UK Clinical Practice Research Datalink showed that those in the most deprived quintile had the highest proportion of multiple morbidity and deaths [24]. Furthermore, a recent large cross-sectional study examining over 1.7 million patients (all ages) using a primary care database in Scotland showed that people living in the most deprived areas tended to develop multimorbidity at a younger age compared to those in affluent areas [2]. Polypharmacy has consistently been shown to be the strongest predictor of PIP, and socio-economic status has been linked to the number of regular medicines prescribed. A large cross-sectional analysis of Scottish primary care patients aged 20 years and over demonstrated a linear relationship between deprivation and number of regular medicines, independent of age group and number of clinical conditions [25]. A nationally representative study on ageing in the Republic of Ireland found a larger difference in prevalence of polypharmacy across levels of educational attainment in people aged 50–64 years compared to those aged 65 and over, indicating socio-economic status may be a more important predictor of polypharmacy in middle-aged individuals than in older people [26]. Deprivation has also been linked with other forms of sub-optimal prescribing, such as higher levels of interacting drugs and lower levels of secondary prevention in older people [27] and high-risk prescribing in adults across all age groups [28].

Although the different socio-economic profiles of the two study populations was likely the biggest contributing factor to differences in PIP prevalence, there may be other cultural factors (potentially influencing the behaviour of the public and doctors) or systems factors (such as government regulation) which shape prescribing practice and give rise to differences between the two jurisdictions [29]. For example, differences between Northern Ireland and the Republic of Ireland in how the healthcare systems are structured may explain the disparity in PIP prevalence to some degree. However, a previous comparative study found larger differences in the prevalence of PIP between the UK and Northern Ireland, which have similar healthcare systems, than between Northern Ireland and the Republic of Ireland, which have similar population demographics, suggesting the latter may be a more important contributory factor [18].

### Most common instances of PIP

The most common instances of PIP were the use of strong opioids without co-prescription of an osmotic or stimulant laxative, prescription of PPIs above the maintenance dosage for greater than 8 weeks and the use of benzodiazepines for greater than 1 month. These findings are consistent with previous studies which reported PIP in the older population. A retrospective cross-sectional study, which used data from the EPD (between 2009 and 2010), applied a subset of 28 criteria from STOPP [30] to more than 166,000 patients aged ≥70 years and reported similar trends in PIP among the older population (use of PPIs at a maximum therapeutic dose for greater than 8 weeks, NSAIDs for greater than 3 months and benzodiazepines for greater than 1 month being the most prevalent) [14]. Similarly, a retrospective national population study using the HSE-PCRS (in 2007), applied 30 STOPP criteria to dispensing records for over 338,000 patients aged ≥70 years with the same PIP criteria occurring most commonly [31].

### Association between PIP, polypharmacy, gender and age

In our study populations, age, gender and polypharmacy were all significantly associated with PIP. There is some evidence of an association between female gender and increased PIP in older people, but this has not been reported consistently [14, 31]. Rates of multimorbidity have been shown to be higher in females than males, which may result in the use of a higher numbers of medications [2]. After controlling for age group and gender, the risk of PIP increased with polypharmacy, identifying polypharmacy as the principal determinant of PIP. This strong association between PIP (as measured by PROMPT) and polypharmacy in this study has also been shown among the older population [14, 31].

Similarities in PIP between middle-aged and older people may suggest that interventions aimed at improving inappropriate prescribing could include both age groups. As a number of the most prevalent PIP criteria related to long-term use, ensuring new prescriptions are reviewed after suitable intervals and discontinued where appropriate may help to address these prescribing issues. Thus far, studies aimed at improving appropriate polypharmacy in older patients have shown some evidence of a reduction in PIP, but any improvement in clinically significant outcomes, such as hospital admissions, medication-related problems and patients’ overall quality of life remains unclear [19]. Future studies investigating healthcare outcomes may help inform interventions to improve prescribing practices in these age groups, particularly in females and the most socio-economically deprived populations where the risk of PIP is elevated. By targeting the ageing population of the future (i.e. the middle-aged), this is a group which will be the focus for health provision in the future.

### Strengths and limitations of this study

This large observational study has several key strengths. Firstly, the PROMPT criteria have been developed for use in prescribing or dispensing datasets in the absence of clinical information, meaning all 22 PROMPT criteria have been applied to the datasets in the North and Republic of Ireland [8]. To our knowledge, this is the first study to determine the prevalence of PIP in middle-aged people. This study demonstrated that the PROMPT criteria are applicable to prescribing in two different health systems. The use of data from the EPD in this study is representative of the entire middle-aged population of Northern Ireland. Conversely, the use of data from the HSE-PCRS GMS scheme represents the most socio-economically deprived third of the middle-aged population of Republic of Ireland. Therefore, the populations in these datasets are not directly comparable. However, this work provides an opportunity to explore how PIP prevalence varies across two similar populations with different healthcare systems and socio-economic profiles. Operational differences between the two datasets used may also have contributed to contrasting prevalence estimates. Medications were classified according to two different systems (BNF and ATC) necessitating a change in coding to ensure comparable analysis of PIP. This may also lead to differences in polypharmacy rates, as BNF categories and the first five characters of ATC codes were used to distinguish drug classes in the EPD and HSE-PCRS database respectively. Differences in medicines used in each jurisdiction may have affected prevalence estimates, for example only one stimulant laxative is available on the HSE-PCRS GMS scheme and hence no cases of this duplication were found.

There are some limitations associated with the use of large dispensing datasets for observational studies. Firstly, in the EPD dataset, approximately 85–90 % of all prescriptions forwarded to the BSO are added to the EPD database [14]. Therefore, a small proportion of prescriptions dispensed by community pharmacies in Northern Ireland will not be collated in this database. Secondly, although this study is only concerned with medications prescribed by a GP, lack of available information on over-the-counter medication use in the datasets could affect the accuracy of prevalence estimates. For example, PIP may be overestimated if a patient on a strong opioid has purchased an over-the-counter laxative, or alternatively may be underestimated if a patient is taking over-the-counter omeprazole while on clopidogrel. Although both of the datasets used longitudinal records of dispensed medications, which eliminates the issue of primary non-adherence to therapies which can occur when prescribing datasets are used, it is still not possible to determine whether patients take (adhere to) medications that have been dispensed. Thirdly, application of the PROMPT criteria to these datasets required a number of assumptions, such as: nitrofurantoin was being prescribed in all cases for uncomplicated lower urinary tract infections, and alpha adrenoreceptor blockers were being prescribed for hypertension only. Finally, as described in the methods, clinical circumstances such as disease diagnosis and duration of medication prescription had to be operationalised for analysis using information included in these large dispensing datasets. Although less accurate than if full patient medical notes with diagnoses had been available, these approaches were consistent with similar administrative database studies [31].

## Conclusion

This study has shown that PIP is prevalent in middle-aged people, a population which was previously under-researched. Further studies investigating healthcare outcomes (such as hospital admissions, medication-related problems) may help inform interventions to improve prescribing practices in this age group, particularly in the most socio-economically deprived populations where the risk of PIP is greatest.

## Electronic Supplementary Material

Supplementary file 1 Analytical approach for different categories of criteria (DOCX 17.6 kb)

Supplementary file 2 Prevalence of all PROMPT criteria (DOCX 30 kb)

## Abbreviations

ATC: Anatomical therapeutic chemical
BNF: British national formulary
BSO: Business services organisation
CIs: Confidence intervals
DDD: Defined daily doses
EPD: Enhanced prescribing database
GP: General practitioner
HSC: Health and social care
HSE-PCRS: Health services executive primary care reimbursement service
HCPs: Healthcare professionals
NHS: National health service
NSAID: Non-steroidal anti-inflammatory drug
ORs: Odds ratios
PIP: Potentially inappropriate prescribing
PROMPT: Prescribing optimally in middle-aged people’s treatments
PPIs: Proton pump inhibitors
TCAs: Tricyclic antidepressants
