# Supplementary material for: Potentially inappropriate prescribing in two populations with differing socio-economic profiles: a cross-sectional database study using the PROMPT criteria
Source: Eur J Clin Pharmacol. 2016 Jan 28;72:583–91. doi: 10.1007/s00228-015-2003-z (PMC4834102; doi:10.1007/s00228-015-2003-z)
Supplement: Supplementary file 1 — Analytical approach for different categories of criteria (DOCX 17.6 kb) [file 228_2015_2003_MOESM1_ESM.docx]

**Title: Potentially inappropriate prescribing in two populations with differing socio-economic profiles: a cross-sectional database study using the PROMPT criteria**

**Journal:** European Journal of Clinical Pharmacology

**Authors:** Janine A. Cooper^a,b*^, Frank Moriarty^b*^, Cristín Ryan^c^, Susan M. Smith^b^, Kathleen Bennett^d^, Tom Fahey^b^, Emma Wallace^b^, Caitriona Cahir^d,e^, David Williams^f^, Mary Teeling^d^, Carmel M. Hughes^a,b^

**Authors’ affiliations:**

^a^Clinical and Practice Research Group, School of Pharmacy, Queen’s University Belfast, 97 Lisburn Road, Belfast, Northern Ireland, BT9 7BL

^b^HRB Centre for Primary Care Research, Division of Population Health Science, Royal College of Surgeons in Ireland, 123 St Stephen’s Green, Dublin 2, Ireland

^c^School of Pharmacy, Royal College of Surgeons in Ireland, 123 St Stephens Green, Dublin 2, Ireland

^d^Department of Pharmacology & Therapeutics, Trinity Centre for Health Sciences, St James Hospital, Dublin 8, Ireland

^e^Economic and Social Research Institute, Whitaker Square, Sir John Roberson’s Quay, Dublin 2, Ireland

^f^Department of Geriatric and Stroke Medicine, Royal College of Surgeons in Ireland, 123 St Stephens Green, Dublin 2, Ireland

**Authorship:** ^*^denotes joint first authorship

**Corresponding author:** Dr. Janine A. Cooper. Queen’s University, Belfast, School of Pharmacy, 97 Lisburn Road, BT9 7BL, Northern Ireland

Telephone: +44 (0) 28 90 97 20 27 | E-mail: [j.cooper@qub.ac.uk](mailto:j.cooper@qub.ac.uk)

**Additional Supporting Information is provided (1 page)**

**Supplementary file 1:** Analytical approach for different categories of criteria

- For criteria that required an assessment of the duration of drug use [for example, a concomitant bisphosphonate should be prescribed if oral corticosteroids are used long-term (greater than three months)], duration of use was assessed using the month a prescription was scanned by the BSO in the EPD dataset and the month of dispensing in the HSE-PCRS database to determine dispensing in consecutive months.
- For criteria referring to a diagnosed condition [for example, Theophylline should not be used as monotherapy for asthma or chronic obstructive pulmonary disease (COPD)]. As no diagnostic information is available in either database, dispensing of any medicine indicated for the treatment of a condition in the BNF was used as a proxy for a diagnosis.
- For criteria required drug dosage information [for example, proton pump inhibitors (PPIs) (e.g. esomeprazole, omeprazole) should not be prescribed at doses above the recommended maintenance dosage for greater than eight weeks], dosage was evaluated by calculating the DDDs using the strength and prescribed quantity of consecutive prescriptions. DDDs are a validated statistical measure of drug consumption maintained by the WHO and may be defined as the “assumed average maintenance dose per day for a drug used for its main indication in adults”.
- The PROMPT criterion ‘Strong opioids (e.g. buprenorphine, diamorphine, fentanyl, morphine, oxycodone) should not be prescribed without the co-prescribing of laxatives’ was analysed by assessing drugs listed as strong opioids in the BNF without the co-prescribing of at least one osmotic or stimulant laxative.
