# Supplementary material for: Potentially inappropriate prescribing in two populations with differing socio-economic profiles: a cross-sectional database study using the PROMPT criteria
Source: Eur J Clin Pharmacol. 2016 Jan 28;72:583–91. doi: 10.1007/s00228-015-2003-z (PMC4834102; doi:10.1007/s00228-015-2003-z)
Supplement: Supplementary file 2 — Prevalence of all PROMPT criteria (DOCX 30 kb) [file 228_2015_2003_MOESM2_ESM.docx]

# Title: Potentially inappropriate prescribing in two populations with differing socio-economic profiles: a cross-sectional database study using the PROMPT criteria

**Journal:** European Journal of Clinical Pharmacology

**Authors:** Janine A. Cooper^a,b*^, Frank Moriarty^b*^, Cristín Ryan^c^, Susan M. Smith^b^, Kathleen Bennett^d^, Tom Fahey^b^, Emma Wallace^b^, Caitriona Cahir^d,e^, David Williams^f^, Mary Teeling^d^, Carmel M. Hughes^a,b^

**Authors’ affiliations:**

^a^Clinical and Practice Research Group, School of Pharmacy, Queen’s University Belfast, 97 Lisburn Road, Belfast, Northern Ireland, BT9 7BL

^b^HRB Centre for Primary Care Research, Division of Population Health Science, Royal College of Surgeons in Ireland, 123 St Stephen’s Green, Dublin 2, Ireland

^c^School of Pharmacy, Royal College of Surgeons in Ireland, 123 St Stephens Green, Dublin 2, Ireland

^d^Department of Pharmacology & Therapeutics, Trinity Centre for Health Sciences, St James Hospital, Dublin 8, Ireland

^e^Economic and Social Research Institute, Whitaker Square, Sir John Roberson’s Quay, Dublin 2, Ireland

^f^Department of Geriatric and Stroke Medicine, Royal College of Surgeons in Ireland, 123 St Stephens Green, Dublin 2, Ireland

**Authorship:** ^*^denotes joint first authorship

**Corresponding author:** Dr. Janine A. Cooper. Queen’s University, Belfast, School of Pharmacy, 97 Lisburn Road, BT9 7BL, Northern Ireland

Telephone: +44 (0) 28 90 97 20 27 | E-mail: [j.cooper@qub.ac.uk](mailto:j.cooper@qub.ac.uk)

**Additional Supporting Information is provided (3 pages)**

**Supplementary file 2: Prevalence of all PROMPT criteria**

**Supplementary file 2:** Prevalence of all PROMPT criteria

|  | **EPD^a^** | | **HSE-PCRS^b^** | |
| --- | --- | --- | --- | --- |
|  | **n** | **% (95% CI)** | **n** | **% (95% CI)** |
| **Gastro-Intestinal System** |  |  |  |  |
| Other than for opioid-induced constipation, stimulant laxatives should not be prescribed as first-line treatment in constipation for > 4 weeks | 291 | 0.1 (0.1, 0.1) | 23 | 0.0 (0.0, 0.0) |
| Proton pump inhibitors (PPIs) should not be prescribed at doses above the recommended maintenance dosage for > 8 weeks | 30,367 | 6.9 (6.8, 6.9) | 54,762 | 17.7 (17.5, 17.8) |
| Esomeprazole or omeprazole should not be used with clopidogrel | 1,795 | 0.4 (0.4, 0.4) | 2,015 | 0.7 (0.6, 0.7) |
| **Cardiovascular System** |  |  |  |  |
| The use of alpha-adrenoceptor blocking drugs as monotherapy for hypertension should be avoided | 2,166 | 0.5 (0.5, 0.5) | 596 | 0.2 (0.2, 0.2) |
| Aspirin doses should not exceed 150 mg/day for anti-platelet therapy | 375 | 0.1 (0.1, 0.1) | 1,448 | 0.5 (0.4, 0.5) |
| Cardio-selective calcium-channel blockers should not be used with beta-adrenoceptor blocking drugs | 543 | 0.1 (0.1, 0.1) | 838 | 0.3 (0.3, 0.3) |
| Oral short-acting dipyridamole should not be used as monotherapy in antiplatelet treatment | 56 | 0.0 (0.0, 0.0) | 74 | 0.0 (0.0, 0.0) |
| **Respiratory System** |  |  |  |  |
| First generation antihistamines should not be used as first-line agents for > 7 days | 11,098 | 2.5 (2.5, 2.6) | 1,566 | 0.5 (0.5, 0.5) |
| A bisphosphonate should be prescribed if oral corticosteroids are used for > 3 months | 1,606 | 0.4 (0.3, 0.4) | 5,756 | 1.9 (1.8, 1.9) |
| Theophylline should not be used as monotherapy for asthma or chronic obstructive pulmonary disease (COPD) | 838 | 0.2 (0.2, 0.2) | 686 | 0.2 (0.2, 0.2) |
| Mucolytic agents should not be used routinely in stable COPD | 80 | 0.0 (0.0, 0.0) | 184 | 0.1 (0.1, 0.1) |
| **Central Nervous System** |  |  |  |  |
| Selective serotonin reuptake inhibitors (SSRIs) should not be used with venlafaxine | 660 | 0.1 (0.1, 0.2) | 927 | 0.3 (0.3, 0.3) |
| Tricyclic antidepressants (TCAs) should not be used as first-line in treatment of depression | 1,176 | 0.3 (0.3, 0.3) | 401 | 0.1 (0.1, 0.1) |
| Benzodiazepines should not be used for > 4 weeks | 12,630 | 2.9 (2.8, 2.9) | 26,395 | 8.5 (8.4, 8.6) |
| Non-benzodiazepine hypnotics should not be used for > 4 weeks | 10,875 | 2.5 (2.4, 2.5) | 25,611 | 8.3 (8.2, 8.4) |
| Carbamazepine should not be used with clarithromycin or erythromycin | 181 | 0.0 (0.0, 0.0) | 422 | 0.1 (0.1, 0.1) |
| Strong opioids should not be prescribed without the co-prescribing of at least one osmotic or stimulant laxative | 30,679 | 6.9 (6.9, 7.0) | 43,041 | 13.9 (13.8, 14.0) |
| **Infections** |  |  |  |  |
| Nitrofurantoin should not be prescribed for > 7 days for uncomplicated lower urinary tract infections | 3,398 | 0.8 (0.7, 0.8) | 3347 | 1.1 (1.0, 1.1) |
| **Endocrine System** |  |  |  |  |
| In diabetes, the use of oral long-acting sulfonylureas should be avoided | 140 | 0.0 (0.0, 0.0) | 90 | 0.0 (0.0, 0.0) |
| **Musculoskeletal System** |  |  |  |  |
| Non-steroidal anti-inflammatory drugs (NSAIDs) should not be used for > 3 months | 6,284 | 1.4 (1.4, 1.5) | 15,488 | 5.0 (4.9, 5.1) |
| Unless Gl protection is provided with PPI/H_2_-receptor antagonist, NSAIDs should not be used in combination with: |  |  |  |  |
| a. Low-dose aspirin. | 3,391 | 0.8 (0.7, 0.8) | 10,170 | 3.3 (3.2, 3.3) |
| b. Selective serotonin re-uptake inhibitors. | 9,840 | 2.2 (2.2, 2.3) | 9,844 | 3.2 (3.1, 3.2) |
| **Duplication of drug classes** |  |  |  |  |
| Benzodiazepines | 5,089 | 1.2 (1.1, 1.2) | 10,539 | 3.4 (3.3, 3.5) |
| Tricyclic antidepressants | 29 | 0.0 (0.0, 0.0) | 211 | 0.1 (0.1, 0.1) |
| Opioids | 16,356 | 3.7 (3.6, 3.8) | 12,523 | 4.0 (4.0, 4.1) |
| NSAIDs | 1,656 | 0.4 (0.4, 0.4) | 11,653 | 3.8 (3.7, 3.8) |
| Loop diuretics | 50 | 0.0 (0.0, 0.0) | 199 | 0.1 (0.1, 0.1) |
| ACE inhibitors | 201 | 0.0 (0.0, 0.1) | 398 | 0.1 (0.1, 0.1) |
| Beta-blockers | 336 | 0.1 (0.1, 0.1) | 764 | 0.2 (0.2, 0.3) |
| Calcium channel blockers | 249 | 0.1 (0.0, 0.1) | 678 | 0.2 (0.2, 0.2) |
| Angiotensin receptor blockers | 48 | 0.0 (0.0, 0.0) | 481 | 0.2 (0.1, 0.2) |
| Statins | 1,245 | 0.3 (0.3, 0.3) | 1,577 | 0.5 (0.5, 0.5) |
| Thiazide diuretics | 72 | 0.0 (0.0, 0.0) | 28 | 0.0 (0.0, 0.0) |
| Non-benzodiazepine (Z-drug) hypnotics | 274 | 0.1 (0.1, 0.1) | 1,319 | 0.4 (0.4, 0.4) |
| Stimulant laxatives | 642 | 0.1 (0.1, 0.2) | 0 | 0.0 (0.0, 0.0) |
| SSRIs | 1,383 | 0.3 (0.3, 0.3) | 860 | 0.3 (0.3, 0.3) |
| Any of the above duplicate drug classes | 25,209 | 5.7 (5.6, 5.8) | 35,105 | 11.3 (11.2, 11.4) |

^a^ Enhanced Prescribing Database, covering the full middle-aged population of Northern Ireland

^b^ Health Service Executive Primary Care Reimbursement database, covering the most socioeconomically deprived third of the middle-aged population of the Republic of Ireland
